# Reconsolidation-based treatment for fear of public speaking: a systematic pilot study using propranolol

**DOI:** 10.1038/s41398-020-0857-z

**Published:** 2020-06-04

**Authors:** James W. B. Elsey, Anna I. Filmer, Harriet R. Galvin, Jennifer D. Kurath, Linos Vossoughi, Linnea S. Thomander, Melissa Zavodnik, Merel Kindt

**Affiliations:** 1 Department of Clinical Psychology, University of Amsterdam, Amsterdam, Netherlands; 2 University Hospital Zurich, Department of Psychiatry, Zurich, Switzerland; 3 Kindt Clinics, Amsterdam, Netherlands

**Keywords:** Psychiatric disorders, Human behaviour, Learning and memory

## Abstract

Pharmacological manipulation of memory reconsolidation opens up promising new avenues for anxiety disorder treatment. However, few studies have directly investigated reconsolidation-based approaches in subclinical or clinical populations, leaving optimal means of fear memory reactivation unknown. We conducted a systematic pilot study to assess whether a reconsolidation-based treatment could tackle public speaking anxiety in a subclinical sample (*N* = 60). As lab studies indicate that the duration of reactivation may be important for inducing reconsolidation, we investigated several speech lengths to help inform further translational efforts. Participants underwent a stress-inducing speech task composed of 3-min preparation, and from 0 to 9 min of public speaking, in 1-min increments. They then received either 40 mg of propranolol (*n* = 40) or placebo (*n* = 20), double-blind, allocated 4:2 for each speech duration. Participants performed a second speech 1 week post treatment, and were followed up with questionnaires 1- and 3 months later. Both self-reported speech distress and questionnaire measures of public speaking anxiety showed clear reductions following treatment. However, propranolol did not reliably outperform placebo, regardless of speech duration at treatment. Physiological responses (heart rate and salivary cortisol) to the public speaking task remained stable from treatment to test. These findings highlight the challenges facing the translation of laboratory research on memory reconsolidation into clinical interventions. Lack of explicit controls for factors beyond duration, such as ‘prediction error’, could explain these null findings, but positive results in clinical interventions are needed to demonstrate that taking such factors into account can deliver the promises of reconsolidation-based therapy.

## Introduction

Recent findings in the neuroscience of learning and memory suggest that, contrary to being immutably etched into the architecture of the brain^1^, emotional memories may be susceptible to change. Since the seminal work of Sara and colleagues^2,3^ and Nader, Schafe and LeDoux^4^, numerous studies over the past two decades have found that reactivation can render a memory vulnerable to interference^5,6^. Based on such findings, researchers have proposed the concept of memory reconsolidation (^3^ cf.^7^): “the reactivation-dependent induction of a transient, unstable state of a previously consolidated memory, during which the memory trace may be modified or disrupted, and requiring a time-dependent process of restabilization in order to persist” (^4,5^ p. 798). Though it remains a contentious issue whether reconsolidation actually occurs in humans or non-human animals^5,8^, the potential clinical implications of reconsolidation have been a cause of considerable excitement^9–11^. If reconsolidation (or some alternative process leading to reactivation-dependent amnesia) can be harnessed and employed in clinical settings, it would be a major breakthrough in the treatment of mental illness, possibly allowing for rapid and long-lasting reductions in symptoms without the need for repeated drug administration or extensive psychological therapy. To date, however, relatively few studies have aimed at translating experimental models of reconsolidation into clinical interventions. In the present experiment, we aimed to assess the feasibility and efficacy of a reconsolidation-based intervention in tackling a naturally occurring fear of public speaking in otherwise healthy young adults. We use the term ‘reconsolidation-based’ to indicate that this intervention is based on the concept of reconsolidation, and not as a definitive statement that reconsolidation underpins any observed effects.

Although behavioural approaches aimed at harnessing reconsolidation are being pursued^12,13^, pharmacological approaches in humans achieve the closest parallels to what we believe are the most convincing demonstrations of retrieval-induced amnesia in animal models, from which reconsolidation was derived. Targeted administration of potent protein synthesis inhibitors used in animals is not feasible for human studies, but well-tolerated, non-toxic drugs, such as the beta-adrenergic receptor antagonist propranolol, can achieve comparable effects^2,14^. It is thought that blockade of adrenergic receptors may affect intracellular signalling pathways that ultimately lead to long-term potentiation—the proposed neural substrate of memory^15,16^.

When administered in time to disrupt reconsolidation, propranolol has proven effective in neutralising conditioned defensive responses in multiple human fear-conditioning studies (e.g., refs. ^17,18^, see ref. ^5^ for a comprehensive review), though not always successful^19,20^. Propranolol has also been the typical drug choice in efforts to alleviate post-traumatic stress disorder (PTSD) with reconsolidation-based procedures^21–23^. These efforts have sometimes been disappointing^24^, but nevertheless highlight the potential of reconsolidation-based pharmacological interventions in tackling strong and naturally occurring emotional memories. However, given that PTSD patients often have highly complex presentations with comorbidity, and great heterogeneity even within PTSD symptoms, PTSD might not be the most instructive disorder to focus translational efforts upon.

While far from simple, more circumscribed anxiety disorders, such as specific phobias, could provide more tractable targets for reconsolidation-based treatments, helping to bridge the gap between experimental models and more complex disorders, while still representing a very strong, durable and naturalistic emotional memory. To reactivate a naturalistic fear memory in spider-fearful participants, Soeter and Kindt^25^ briefly exposed participants to a live tarantula. This ‘memory reactivation’ was immediately followed by oral propranolol administration. Participants treated with propranolol + reactivation showed dramatic reductions in fear of spiders, and were typically able to touch or even hold spiders at least up to 1 year after the intervention. In contrast, those receiving placebo + reactivation or propranolol alone showed no changes in their fear. These control conditions demonstrate that the fear reduction cannot be explained by a general fear-dampening effect of propranolol, or by mere exposure. Similar effects have been reported in case studies of other animal phobias^26^, but we are not aware of controlled studies of pharmacological reconsolidation-based treatments for specific fears or phobias since then. In the present experiment, we aimed to extend this approach to a circumscribed social fear: fear of public speaking.

Fear of public speaking is a ‘performance only’ subtype of social anxiety disorder (SAD), characterised by extreme fear in, and avoidance of, public speaking situations, without more general social impairment as a result of anxiety^27^. Relative to pervasive SAD, those who specifically fear public speaking typically develop their fear later, may have less comorbid issues and personality problems and have lower genetic risk^28–30^. Public speaking can provide a good test case for reconsolidation-based treatments, as the feared object/situation is clearly different from animal phobias, yet sufficiently similar in the form of anxiety response and proposed aetiology as to not reflect a massive stretch beyond existing applications. Furthermore, fear of public speaking is itself a worthy target of novel interventions. It is one of the most common fears and, in the extreme, can result in missed educational, social and workplace opportunities^31^. Taken together, these considerations suggest that public speaking anxiety could be both a valuable and informative target for the translation of reconsolidation-based interventions.

One major difficulty for the translation of reconsolidation-based treatments is that the optimal means of reactivation—that is, in what manner feared stimuli are represented to participants, or the way in which participants are required to confront their fears—are not well-understood. Reactivation does not always cause reconsolidation, and may instead lead to mere retrieval, or to the generation of a new memory trace. Experimental studies suggest that something beyond merely representing the feared stimulus may be needed to render memory vulnerable to interference. Several animal^32^ and human^33,34^ fear-conditioning studies suggest that reconsolidation may only be triggered when reactivation includes ‘prediction error’: that is, the reactivation violates some expectation learned during the initial conditioning procedure (e.g., the presence or magnitude of the learned outcome differs from expectations). Yet, if multiple prediction errors occur, then extinction is likely to be triggered^35,36^. Studies also suggest that a limbo phase lies between reconsolidation and extinction: at some moderate level of prediction error, neither reconsolidation nor extinction are induced, and the memory simply remains stable^35,36^. The duration of reactivation has also been suggested as a key ingredient for triggering reconsolidation. In some cases, it appears that duration is relevant insofar as it allows for varying degrees of prediction error to occur^37^, whereas others suggest that duration itself is key, regardless of prediction error^38^. Similarly to prediction error findings, brief reactivations typically induce reconsolidation, long reactivations (20–30 min) provoke extinction and a ‘limbo’ phase lies in between. What length of reactivation is desirable in different clinical contexts is unknown. Although we would not expect any single form of reactivation to always trigger reconsolidation for everyone, the experimental studies above suggest that certain means of reactivation can be more or less effective in triggering reconsolidation (i.e., reactivations that provide an opportunity for prediction error, and/or are not of especially long duration).

As a first attempt in public speaking, we aimed for a standardised and easily replicable procedure, using the well-studied Trier Social Stress Test (TSST)^39^ to provoke social-evaluative fear. For this pilot investigation, we systematically varied the length of reactivation, in the hope of identifying what (if any) length of reactivation might be most productive to focus on in a larger randomised controlled trial. Drawing on the success of Soeter and Kindt’s^25^ spider study, in which reactivation-dependent amnesia was observed by combining a relatively simple and brief fear-provoking spider confrontation with propranolol administration, we did not directly manipulate prediction error. Instead, we encouraged participants to drop some safety behaviours, such as seeking reassurance from the audience, and to try to continue to talk even if they felt they were ‘blanking’. As with a frightening confrontation with a spider, this type of reactivation can provide many opportunities for prediction error occurrence, such as the expectation that the audience will laugh, or that one will have a panic attack (limitations of this approach will be considered in the ‘Discussion’).

**Fig. 1:**
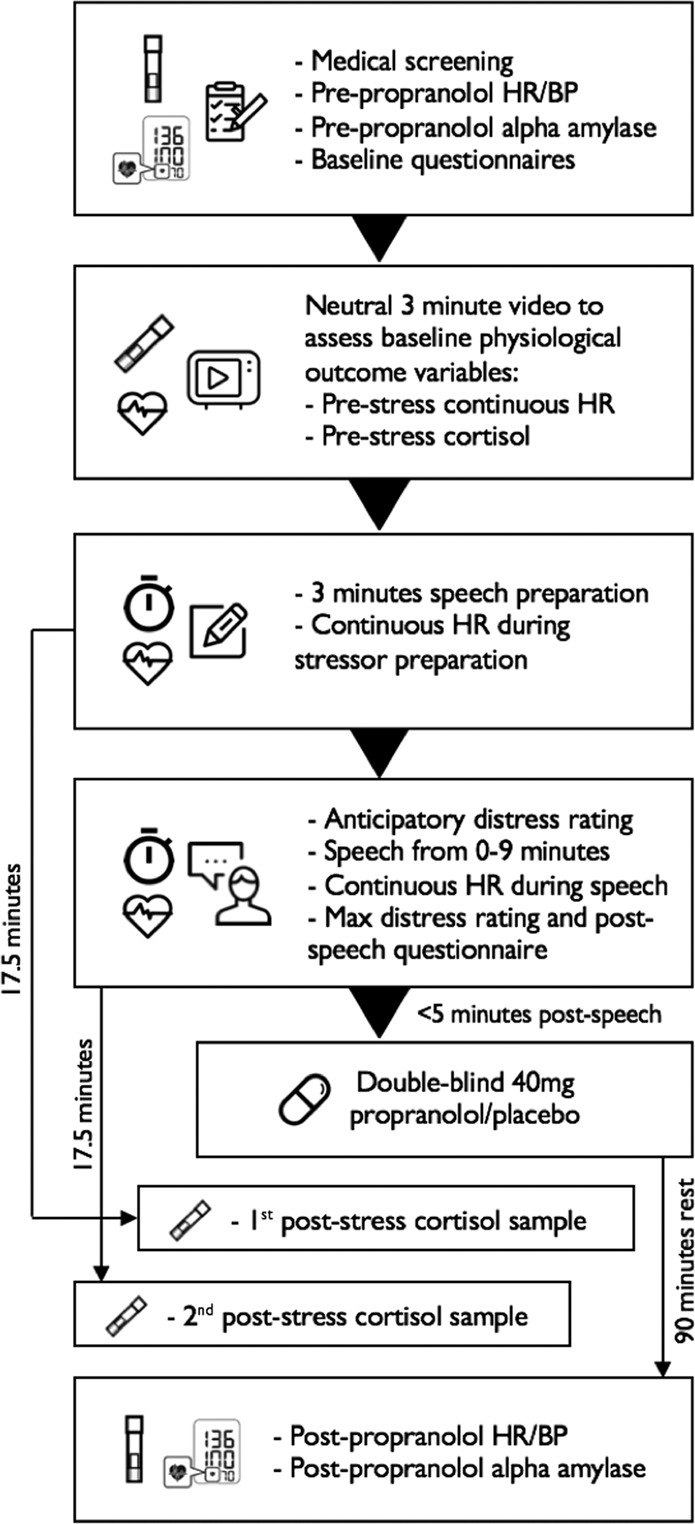
Experimental procedure for Session 1. BP Blood pressure, HR Heart rate.

In brief, participants with high fear of public speaking were required to undergo a public speaking task of variable duration, and then received either 40 mg of oral propranolol or placebo, so as to disrupt the putative post-reactivation process of reconsolidation. A diagram of the experimental procedure is provided in Fig. 1. Participants undertook another speech 1 week later to assess treatment effects, and were followed up by a questionnaire 1- and 3 months later to assess longer-term impact. The primary outcome variables were a questionnaire measure of public speaking anxiety, as well as self-reported distress induced by the public speaking challenge, and self-rated speech performance. Stress-induced changes in cortisol and heart rate were also assessed in Session 1 (S1) vs. Session 2 (S2). Details of all procedures, measures and the analytic approach, are provided in the ‘Methods' section. To our knowledge, this is the first attempt to tackle fear of public speaking using a reconsolidation-based approach, and can provide useful guidance for further controlled attempts to tackle this and other fears in future experiments.

## Results

### Manipulation check

Analysis of alpha amylase, heart rate (HR) and blood pressure (BP) measured at the beginning of S1 and 90 min after pill ingestion generally indicated a successful effect of propranolol, whereby declines over time were greater for the propranolol relative to the placebo group, with the exception of diastolic blood pressure. Bayes Factors for the inclusion (*BF*_Inclusion_) of a Condition*Time interaction were 104.22, 61.39, 8.90 and 0.85, for alpha amylase (log-transformed), HR, BP_Systolic_ and BP_Diastolic_, respectively. A means table is presented in the Supplementary Materials. State anxiety decreased over this time (*BF*_Inclusion_Time_ = 2.49e + 13), but decreases did not vary by Condition (*BF*_Inclusion_Pill*Time _= 0.66). Hence, propranolol affected physiology but not subjective anxiety.

### Baseline measures

Bayesian *t*- and Mann–Whitney *U* tests indicated no confounds in baseline variables between groups (Table 1). There was also no evidence for correlations (Pearson’s *r* and Kendall’s *tau*) between Duration and any baseline measure (only the Liebowitz Social Anxiety Scale—Fear section [LSAS_Fear_] showed weak evidence). The additional single items indicate that participants tended to experience the speech as slightly worse than expected, and tended to ruminate over the experience afterwards. Responses to these items did not differ between groups (all *BF*_10_ < 0.4). There was a clear tendency for longer durations to produce greater rumination. There was only slight and inconsistent evidence that Rumination might relate to change in some main outcome variables (Rumination_Wait_ marginally associated with greater change in Global Perception of Speech Performance [GPSP]: tau = −0.22, *BF*_10_ = 2.53; Rumination_Day_ marginally associated with less change in Personal Report of Public Speaking Anxiety [PRPSA]: tau = 0.21, *BF*_10_ = 2.82). Sex was proportionally distributed across propranolol (33 females) and placebo groups (17 females) (*BF*_10_ for independent multinomial contingency test = 0.25). Given these findings from baseline variables, we did not consider any as confounds for the main analyses.

**Table 1:** Baseline characteristics of participants by group.

	Mean (SD)		*BF*_10_			Duration	
	Prop	Placebo	*t*		*U*	*r (BF*_10_*)*	*tau (BF*_10_*)*
*Baseline measures*							
Age	21.65 (2.78)	22.10 (1.92)	0.33	0.34		0.01 (0.16)	0.02 (0.17)
LSAS_Avoid_	21.35 (9.66)	21.35 (9.60)	0.28	0.29		0.20 (0.53)	0.13 (0.50)
LSAS_Fear_	27.63 (9.81)	25.65 (10.54)	0.34	0.36		0.28 (1.59)	0.19 (1.53)
ASI	17.05 (8.11)	18.10 (9.42)	0.30	0.28		−0.04 (0.17)	−0.03 (0.17)
PRPSA	138.33 (9.20)	138.70 (9.49)	0.28	0.28		0.09 (0.20)	0.03 (0.18)
STAI-S	43.55 (8.61)	40.25 (9.68)	0.58	0.62		0.05 (0.17)	0.04 (0.18)
STAI-T	43.30 (6.98)	45.50 (11.71)	0.39	0.64		−0.04 (0.17)	−0.04 (0.18)
PHQ-9	4.00 (2.62)	3.95 (2.65)	0.28	0.26		0.12 (0.25)	0.10 (0.31)
RSES	19.20 (3.84)	19.70 (5.45)	0.30	0.32		−0.10 (0.21)	−0.07 (0.23)
STAI-S S2	38.65 (10.77)	41.05 (10.75)	0.36	0.36		NA	NA
*Additional single items*							
Confidence	6.15 (1.72)	6.25 (1.94)	0.28	0.32		−0.03 (0.16)	−0.02 (0.17)
Rumination_Wait_	55.80 (25.36)	61.95 (24.00)	0.39	0.32		0.46 (123.92)	0.32 (95.32)
Rumination_Day_	42.93 (24.77)	38.75 (24.88)	0.32	0.35		0.33 (3.72)	0.22 (3.38)
Versus expected	−17.78 (45.38)	−21.53 (41.82)	0.30	0.29		−0.11 (0.23)	−0.06 (0.22)
Versus expected_Absolute Score_	39.17 (28.40)	37.88 (26.77)	0.30	0.34		0.01 (0.17)	−0.02 (0.18)

*BF*_10_ Bayes Factor for difference between groups/relationship with Duration, *prop* Propranolol group, *r* Pearson’s *r*, *SD* standard deviation, *t* Bayesian independent samples’ *t* test, *tau* Kendall’s *tau,* *U* Bayesian Mann–Whitney *U* test, *LSAS* Liebowitz Social Anxiety Scale, *ASI* anxiety sensitivity index, *PRPSA* personal report of public speaking anxiety, *STAI-S/T* state-trait anxiety inventory—state/trait, *PHQ-9* patient health questionnaire 9, *RSES* Rosenberg Self-esteem Scale.

### Self-report outcome measures

**Fig. 2:**
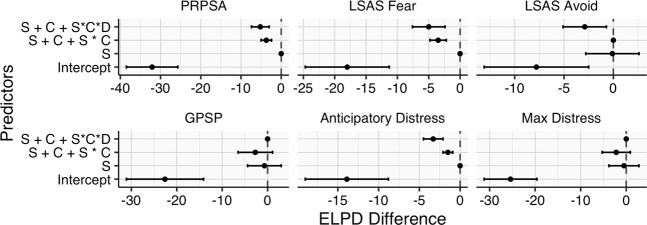
Leave-one-out cross-validation for each primary and secondary outcome variable indicates that inclusion of Session as a predictor typically improves model performance, with no benefit of other predictors. ELPD expected log pointwise predictive density vs. best model, S session, C condition, D duration, *interaction between predictors.

Posterior parameter estimates for each model are fully presented in the Supplementary Materials. Candidate models for predicting primary and secondary outcome variables were assessed with Pareto Smoothed Importance Sampling Leave-One-Out Cross Validation (PSIS-LOO)^40^. In brief, LOO cross-validation repeatedly leaves out individual datapoints when estimating model parameters, then assesses the models’ errors in predicting each left-out point. In doing so, it aims to account for possible model overfitting. The output of this process is estimated by PSIS-LOO. The key output of PSIS-LOO is the difference in Expected Log Pointwise predictive Density (ELPD Difference). Relative to the best-performing model—set to ‘0’—models that perform worse in cross-validation will have reliably negative ELPD Difference scores. Based on this metric, including Session as a predictor typically improved model performance vs. the Intercept-only model (Fig. 2). For LSAS_Avoid_, models did not convincingly outperform the Intercept alone. Additional predictors (interactions with Condition/Duration) resulted in no or negligible improvement of model performance.

Corroborating these findings, *BF*_Inclusion_ for predictors when the data are analysed in *JASP* in either a two-way (Session by Condition) Bayesian mixed-measure ANOVA, or a linear regression on change scores, with Condition, Duration and their interaction as predictors, overwhelmingly supports an impact of Session for PRPSA (7.08e + 8), Distress_Anticipatory_ (1099.51), Distress_Max_ (2.64e + 8), GPSP (2.54e + 9), LSAS_Fear_ (7424.54) and LSAS_Avoid_ (26.10) (Supplementary Table S3). The results point against inclusion of Condition, Condition*Session, Duration and Duration*Condition (*BF*_Inclusion_<1). Meagre evidence is found for an effect of duration on change in Distress_Max_ (*BF*_Inclusion_ = 2.35), which does not appear to vary by Condition.

**Fig. 3:**
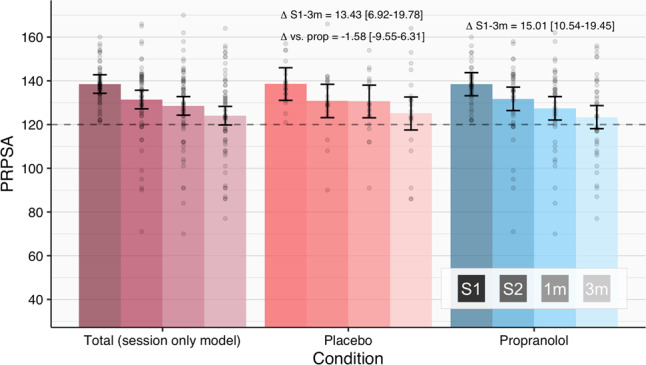
Estimated fitted means (with 95% central posterior density intervals) for PRPSA scores from S1 to 3-month follow-up. Comparison of Placebo and Propranolol groups suggests no benefit of receiving propranolol vs. placebo. Points show raw scores. The dashed line reflects the initial cut-off score for inclusion.

Fitted means (estimated means from the posterior distribution of regression parameters) of the Session*Condition model indicate that PRPSA scores for the propranolol and placebo groups are predicted to decrease from S1 to a 3-month follow-up (3 m), with negligible difference in change between groups (Fig. 3). Change over time may be most parsimoniously explained by Session alone, estimating a drop of 14.48 (18.07–10.81, 95% central posterior density interval [PDI]).

**Fig. 4:**
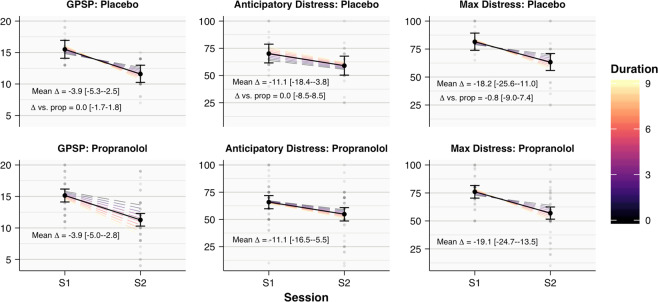
Estimated fitted means (with 95% central posterior density intervals) for GPSP and distress scores from S1 to S2, from the Session*Condition*Duration interaction model, suggest comparable change over time in Placebo and Propranolol participants. The solid black line and points represent mean change across durations. Dashed lines reflect fitted means for each duration. Grey points show raw scores.

Similarly, for GPSP, Distress_Anticipatory_ and Distress_Max_, the estimated average S1–S2 changes from the Session*Condition*Duration model, indicating that scores on all measures are expected to decrease, but this change is not different between groups (Fig. 4). By subtracting estimates of the effect of duration in S2 vs. S1 for each condition, and then comparing these differences, we can also estimate any possible Session*Condition*Duration interaction directly. There is clear evidence against an interaction, with this ‘difference in differences’ estimated at 0.01 (−0.37–0.39, 95% PDI), 0.00 (−1.89–1.88, 95% PDI) and −0.18 (−2.01–1.64, 95% PDI), for GPSP, Distress_Anticipatory_ and Distress_Max_, respectively. The favoured Session-only model predicts declines of 3.95 (2.92–4.95, 95% PDI), 11.10 (6.46–15.78, 95% PDI) and 19.45 (14.63–24.38, 95% PDI), for these variables. For the secondary self-report outcome variables of LSAS_Fear_ and LSAS_Avoid_, the favoured Session-only model predicts modest drops in scores from S1 to 3-m follow-up of 4.41 (2.60–5.06, 95% PDI) and 2.66 (0.84–4.46, 95% PDI).

### Physiological measures

No treatment-related effects were apparent for physiological measures. For HR, including Timepoint, improved model predictions vs. Intercept alone (ELPD_Difference_ = −253.3, SE = 14.3). Including interactions with Session (ELPD_Difference_ = −1.1, SE = 1.9) or Session and Condition (ELPD_Difference_ = −3.7, SE = 3.4) provided no improvement. The time-point model simply predicts baseline HR at 72.61bpm (95% PDI = 69.32–75.81), increasing by 20.06bpm (95% PDI = 17.66–22.51) during speech preparation, and 42.07bpm (95% PDI = 39.50–44.59) during the first minute of speech, irrespective of session. Analysis as a mixed-measure ANOVA in JASP likewise indicated overwhelming evidence favouring inclusion of Timepoint (*BF*_Inclusion_ = 1.61e + 15), with clear evidence against Session*Timepoint*Condition (*BF*_Inclusion_ = 0.01).

For log-cortisol responses, no models convincingly outperformed the Intercept alone in PSIS-LOO cross-validation (vs. Timepoint*Session*Condition model: Intercept ELPD_Difference_ = −7.8, SE = 5.8, Timepoint ELPD_Difference_ = −1.5, SE = 4.0, Session*Timepoint ELPD_Difference_ = −2.0, SE = 3.8). For the treatment-relevant model involving a Timepoint*Session*Condition interaction, all parameters’ 95% PDIs, excluding the Intercept, spanned 0, indicating insufficient evidence of an effect of any predictor. Analysing log-cortisol in JASP as a mixed-measure ANOVA similarly failed to provide evidence for any treatment effect (Timepoint*Session*Condition *BF*_Inclusion_ = 0.56), with a weak indication that the propranolol group’s cortisol levels might have been greater in S2 (Session*Condition *BF*_Inclusion_ = 2.09).

## Discussion

Our study aimed to assess the feasibility of tackling fear of public speaking using a pharmacological, reconsolidation-based intervention. Taken together, our findings indicate that participants experienced a moderate decline in fear of public speaking from S1 to S2, and further general improvement in questionnaire measures of public speaking anxiety at 1- and 3-month follow-ups. Physiological measures indicated that propranolol exerted its expected influence over beta-adrenergic activity. However, changes in public speaking anxiety were not contingent upon receiving propranolol. We would therefore suggest that well-known phenomena such as placebo effects (e.g., expecting one’s fear to decrease allowing one to become more confident), or practice/exposure effects (e.g., being familiar with the task at the second performance, or practicing a speech under difficult experimental circumstances increasing one’s confidence when speaking with a more receptive audience outside of the study), underpin the observed anxiety reductions, rather than any novel phenomena such as reconsolidation. These findings contrast with previous results in spider-fearful participants^25^, where fear levels of control participants remained stable, and rapid and substantial decreases in fear of spiders were observed in reactivation + propranolol participants. Physiological responses to the stressor were not affected by the treatment.

In addition to manipulating whether participants received propranolol or placebo, we varied the duration of reactivation. Only maximal distress indicated a possible influence of speech duration (not varying with Condition), with participants who performed longer speeches experiencing greater declines in distress. As participants gave this rating *after* their speeches, this could be an artefact of the shorter duration of test-session speeches vs. treatment for those participants receiving 5–9-min reactivations. Shorter test speeches may have relieved these participants, who might have anticipated longer talks. Although we cannot strictly eliminate the possibility that longer speeches could produce reconsolidation-like effects, this seems unlikely given the effectiveness of much shorter reactivations in Soeter and Kindt^25^, clinical case observations and lab experiments. We therefore tentatively suggest that the reactivation employed may be ineffective in triggering reconsolidation.

While in Soeter and Kindt^25^, a brief fear-provoking exposure appears to have been sufficient to trigger reconsolidation, inducing reconsolidation is a delicate balancing act involving learning history, prediction error and possibly duration and other factors. Some participants may have found that the modified TSST confirmed their fears (it was slightly worse than expected, on average), as panel members provided no feedback. In addition, it should be considered that a substantial part of public speaking anxiety is both anticipatory and retrospective (e.g., post-event rumination)^41,42^. Participants did appear to ruminate on the experience afterwards, and might also have begun feeling anxious in anticipation of the task. Given these possibilities, one could consider giving positive feedback to participants, which may help both to provide some form of prediction error (an unambiguously positive response) and to curb negative post-event processing (due to satisfaction with one’s performance). It could also be that the TSST situation is too contrived to render a naturalistic fear memory vulnerable to interference (a difficulty that may be insurmountable if participants’ core fears involve failing classes or being ostracised by their peers, rather than the speaking scenario itself). Using a more realistic speech setting with more audience members, but without them having to maintain neutral expressions, is also possible, as well as requiring participants to give their speeches unexpectedly.

However, we remain largely ignorant of the parameters causing successful reconsolidation-based interventions for naturalistic and clinical fears. Prediction error can be easily operationalised in experimental studies where learning and reactivation are precisely controlled, but not in naturalistic fears. People with specific fears can express a wide range of expectations related to their fear, and it is not clear which—if any—should be focused on in an intervention. Nevertheless, our findings do suggest that merely provoking social-evaluative anxiety in individuals with fear of public speaking is unlikely to be sufficient for inducing reconsolidation.

Though we have focused on the idea that the current means of fear memory reactivation did not induce reconsolidation as the most likely explanation for the null effects, it could also be considered whether the pharmacological manipulation itself, or its timing, may be at fault. Research in fear-conditioning paradigms from our lab has consistently found 40 mg of propranolol to be an effective dosage for fear neutralisation, irrespective of participant body mass^43^, and that the drug can be administered up to 1 h post reactivation^18^. Administering 40 mg of propranolol shortly after reactivation was also effective in tackling a long-standing fear of spiders^25^, and in reducing PTSD symptoms in a case series^23^. However, recent research suggests that factors, such as the learning context’s familiarity and the strength of learning, can affect the timing of the consolidation window^44^. Different tasks might similarly affect the reconsolidation window, rendering our drug delivery approach suboptimal. Given this possibility, one option for future experiments might be to administer propranolol 30–45 min before reactivation, meaning that it would be unlikely to have any subjective effects during reactivation, but would be physiologically active more rapidly afterwards. Alternative drugs altogether have also been investigated for their reconsolidation-disrupting potential in clinical settings^45^.

In conclusion, this systematic pilot study did not achieve reconsolidation-like effects for public speaking anxiety. Consideration of why this was unsuccessful can be instructive. Although we suggest that alternative means of reactivating participants’ social-evaluative fears could prove more fruitful, it remains possible that such anxiety disorders are not amenable to pharmacological reconsolidation-based procedures. The outcomes of placebo participants in this study also emphasise the importance of including control participants in studies of reconsolidation-based interventions, as it cannot be assumed that non-specific changes in clinically relevant measures will not occur even with very short interventions. Finally, our findings highlight that despite the great promise of reconsolidation-based interventions, clinical translation is highly complex.

## Materials and methods

### Participants

Participants were recruited via campus flyers and online advertisements, which linked to an online-screening questionnaire. Potentially eligible participants underwent a telephone screening, and an in-person blood pressure/heart rate (BP/HR) check if these values were uncertain. Included participants were required to be medically fit to receive a 40-mg dose of propranolol (for full criteria, see Supplementary Materials), have a Patient Health Questionnaire (PHQ-9)^46^ score <10 and Personal Report of Public Speaking Anxiety (PRPSA)^47^ score of ≥120 at screening and the first study session (S1). According to McCroskey’s^47^ norms, 120 represents the high end of ‘moderate’ anxiety, with 134 rated as ‘high’. This slightly lower cut-off was based on assessing pilot participants, who displayed high anxiety but not always extreme PRPSA scores. For comparison, PRPSA scores of high-fear participants undergoing an exposure intervention for public speaking anxiety by another research group averaged at 133.2, with approximately half of participants scoring in the ‘moderate’ range, and half in the ‘high’ range^48^. Average scores in our sample were 138–139. Telephone screenings further ensured that participants were highly anxious about public speaking. Additional criteria were fluency in English as a second language, being aged between 18 and 28, current enrolment in a bachelor’s or master’s programme, not reporting any mental health issues besides fear of public speaking and not undergoing any other mental health treatment.

During telephone screening, participants underwent a Structured Clinical Interview for DSM-5 (SCID) social anxiety disorder, determining that participants were *not* experiencing clinical social anxiety outside of public speaking situations, and that they *were* experiencing clinically significant anxiety related to public speaking situations. Interviewers (INITIALS_BLINDED_FOR_REVIEW) were trained in administration of this SCID-5 section by the first author, who underwent group training by Dr. Michael First. DSM requirements were relaxed for some criteria. Specifically, in our experience, most anxious participants have ‘cognitive insight’ that their fears are not rational/justified, but nevertheless suffer from severe anxiety. Participants who recognised that they would not suffer disproportionate negative social consequences due to poor speech performance (item F33), but who still experienced consistent and severe public speaking anxiety, were considered to have public speaking anxiety. Secondly, as students only intermittently face public speaking situations and cannot be expected to suffer daily from this fear, we considered interference surrounding a public speaking event, rather than daily interference. Hence, participants may be described as having a subclinical, circumscribed social anxiety.

In a minority of exceptional cases (*n* = 6), S1 speech panel members suggested exclusion of a participant who otherwise met inclusion criteria because they either had failed to perform the speech task with sufficient seriousness or did not appear legitimately anxious. These exclusion decisions were made before participants returned for the second study session (S2). Supplementary Materials include a flow diagram indicating all reasons for exclusion at different stages of this pilot.

Sixty participants (50 female) aged 18–28 (mean = 21.80, SD = 2.52) were included. These 60 include a 3-min propranolol participant who did not want to return for their second speech, but completed S2 questionnaires online. The final sample included 40 propranolol and 20 placebo participants. The initial design included only 10 placebo participants, who intended to ensure that experimenter’s attitudes did not change dramatically if a fully placebo-controlled trial followed this pilot. Observation of unexpected placebo effects in another study prompted the inclusion of 10 more placebo participants after our pilot had commenced, in order to estimate possible placebo effects. Participants received €40/4 credits for participation, plus €5/.5 credits for 3-month follow-ups. All procedures were approved by the University of Amsterdam ethics review board under code 2016-CP-7282, and all participants gave informed consent.

### Propranolol

Propranolol (40 mg) was administered orally in pill form, within 5 min of speech termination in S1. Propranolol pills were made by Accord Healthcare Ltd. (UK), and provided along with placebo pills by Huygens Apothecary (NL). This dosage has been effective in multiple experimental reconsolidation studies, irrespective of participant body mass^43^, and also effectively used in tackling another subclinical, naturalistic fear^25^, as well as in a case series of patients with PTSD^23^. Two department members pseudorandomly allocated participants to receive either propranolol (*n* = 40) or placebo (*n* = 20). Each duration from 0 to 9 min was allocated six participants with a 4:2 ratio of propranolol:placebo. Pills were administered double-blind. Given the uneven propranolol:placebo ratio, analyses were not blind.

### Materials and measures

Several validated self-report measures (see Supplementary Materials for psychometric properties) were used to assess baseline participant characteristics and change over time.

### Primary outcome measures

Public speaking anxiety was assessed using the PRPSA^47^. Scores on this 34-item self-report scale range from 34 to 170, with higher scores indicating greater speech anxiety.

Anxiety experienced while performing the public speaking task was assessed using Subjective Units of Distress/Discomfort (SUDS)^49^. Participants rated their distress from 0 (no distress) to 100 (extreme distress) at two occasions in each in-person session: once immediately before entering the speech room (Distress_Anticipatory_), and once to report their maximal distress after exiting the speech room (Distress_Max_). Participants were familiarised with the meaning and use of the SUDS in the pre-task interview period. The participant who did not return for their S2 speech received distress scores of 100.

Participants’ impressions of their public speaking performance (e.g., ‘Appeared confident’) were assessed using the Global Perception of Speech Performance-Self-rating (GPSP)^50^. Sum scores on this 5-item self-report scale can range from 0 to 20. Higher scores indicate poorer perceived performance.

### Secondary outcome and baseline measures

The Liebowitz Social Anxiety Scale (LSAS)^51,52^ was included as a secondary outcome measure to determine if any changes in fear of public speaking might also extend to a generalised social anxiety measure. Notably, scores cannot completely disambiguate fear in public speaking vs. other performance/general social situations, and so any reductions in the total score should be considered a tentative indication of possible general social anxiety effects. Scores on the 24-item *LSAS*_Fear_ and *LSAS*_Avoid_ subscales of this self-report measure can range from 0 to 72. Higher scores indicate greater anxiety in or avoidance of several social situations. Scores combining both subscales in our sample averaged below 50, whereas the average of those diagnosed with or undergoing treatment for general social anxiety typically stand between 70 and 80^53,54^, consistent with our intention to recruit those with more circumscribed/subclinical social anxiety.

The PHQ-9^46^ was used to screen out participants experiencing ‘moderate’ or greater depressive symptoms (i.e., scoring ≥10). Scores on this 9-item self-report scale can range from 0 to 27. Higher scores indicate more depressive symptoms over the past 2 weeks.

Baseline assessments were also made for anxiety sensitivity, self-esteem and state-trait anxiety, using the Anxiety Sensitivity Index (ASI, range = 0–64)^55^, Rosenberg Self-Esteem Scale (RSES, range = 10–40)^56^ and Spielberger State-Trait Anxiety Index (STAI, range = 20–80)^57^. Higher scores on these self-report scales respectively indicate higher anxiety sensitivity, self-esteem and state/trait anxiety. Additional STAI-State measurement at the end of S1 enabled assessment of whether propranolol affected participants’ subjective anxiety.

Four single-item measures were included to gain insight into participants’ experience of the procedure for future designs. Participants indicated their confidence in the treatment approach in S1, from 0 (‘none at all’) to 10 (‘complete confidence’). As a rough index of prediction error, participants used a sliding scale to indicate their experience of the task relative to expectations, from −100 (‘much worse’), through 0 (‘as expected’), to +100 (‘much better’). At the conclusion of S2, participants used two sliding scales to retrospectively report how much they ruminated over their first speech during the waiting period (Rumination_Wait_) and the rest of the day (Rumination_Day_), from 0 (not at all) to 100 (constantly).

### SCID-5 social anxiety

Participants underwent an assessment of social and public speaking anxiety using the SCID-5 social anxiety section, covering diagnostic criteria for social anxiety disorder.

### Physiological measures

#### Propranolol manipulation checks

To confirm propranolol’s adrenergic influence, participants gave two saliva samples (using ‘Code Blue’ Sarstedt Salivettes, Germany)—first at the beginning of S1 during the initial medical screening, then 90 min after pill ingestion—which were assessed for alpha amylase content. Blood pressure and heart rate (BP/HR) were also assessed at these times to measure cardiovascular effects of propranolol, using an Omron Corporation (Japan) sphygmomanometer.

#### Stress-related outcome measures

Three cortisol samples were taken per session. The cortisol baseline was taken while participants watched a 3-min nature video segment after baseline questionnaires. Post-stress samples were taken 17.5 min after task preparation began and 17.5 min after speech completion, coinciding with the timing of peak cortisol levels^39^ while factoring in variability from different speech durations. For very short speech durations, the timing for post-stress cortisol samples sometimes overlapped, resulting in one sample being collected. S2 took place 15–45 min after S1 (on another day), to control for daily fluctuations in cortisol. One propranolol participant whose sessions occurred >2 h apart was excluded from cortisol analysis. Samples were collected using an oral salivette (‘Code Blue’ Sarstedt, Germany) and frozen to at least −20 °C within an hour of collection. Quantification of salivary analytes was performed by Dresden LabService GmbH, as detailed in the Supplementary Materials.

Continuous HR measurement using a Polar H10 monitor via *Heart Rate Variability Logger* iOS app^58^ was included as an exploratory measure. HR collected from Polar devices shows near- perfect correlation with electrocardiography^59^. Our analyses used the average HR for the middle 2 min of the pre-speech baseline period (during the nature video), for the 3-min speech preparation period, and for the first minute of their speech. Timepoints required ≥66.67% complete second-by-second measurements for inclusion (six datapoints were excluded).

### Modified TSST

At S1 and S2, participants underwent a modified TSST^39^. Stress induction began as in the typical TSST, with participants instructed that they would deliver a speech to a small audience trained in behavioural analysis, and they should make the best impression possible. A camera would record them for later analysis. Participants were required to pitch themselves as candidates for their ideal job/a competitive study programme (counterbalanced across sessions), and to maintain this role for the entire speech.

One modification to the TSST was intended to limit the use of certain safety behaviours, which can prevent prediction error. Participants were instructed not to seek to alleviate pressure on themselves by asking the audience questions, or by giving up if they thought they were running out of things to say (these instructions are not given in the typical TSST). A further modification was that instead of 5 min of speaking and 5 min of arithmetic as in the typical TSST, participants were informed that they would speak for up to 10 min. S1 speeches ranged from 0 to 9 min, in 1-min increments. This range of durations was intended to allow sufficient time for participants to experience some sort of prediction error (e.g., the audience does not laugh, they do not have a panic attack), but not so long that extinction could be expected to occur. To enable comparison directly across durations at S2, the second speech was kept constant at 4.5 min, reflecting the average of all the other speech lengths, and closely matching the speech length of the usual TSST. Panel members for S2 were blind to the S1 speech duration (except for 0-min participants, in case these participants needed further instruction, which was not ultimately required).

As in the standard TSST, one panel member asked the speaker questions after approximately 3 min. The two panel members were a smartly dressed man and woman, who maintained a neutral demeanour. Panel members changed from S1 to S2 to reduce exposure effects if the exact same audience were present at both speeches. Speeches were terminated by the experimenter knocking on the door from outside the speech room. The panel then thanked the participant and asked them to exit.

### Procedure

*S1*: Figure 1 provides a schematic representation of the S1 protocol, including timing of saliva samples and main events. Sessions began with medical screening, pre-propranolol BP/HR and salivary alpha amylase measurement, and attaching the continuous HR monitor, followed by questionnaire completion. Pre-stress cortisol and continuous HR was then measured. Participants then briefly discussed their first, worst and most recent public speaking situations, during which the SUD scale was explained and utilised to familiarise them with its use. The modified TSST was then performed, with associated SUDs and performance ratings. This was followed by propranolol/placebo administration (double-blind). Post-task questionnaires were very brief, meaning that propranolol was administered within 5 min of speech termination. A 90-min rest period with light reading material, allowing propranolol to reach peak bioavailability^60^, then followed. The two post-stress cortisol samples were taken during this rest period. At the close of the session, participants completed the STAI-S and had their BP/HR and salivary alpha amylase measured again.

*S2*: Test sessions were arranged for between 6 and 9 days after the treatment session, 15–45 min after the S1 time. Participants fitted the HR monitor and completed the STAI-S. Pre-stress cortisol and continuous HR were then assessed. The 4.5-min modified TSST was then performed with associated SUDs and performance ratings. An approximately 20-min rest period took place after the TSST to allow the two post-stress cortisol samples to be taken, after which participants completed the PRPSA and LSAS.

### One- and three-month follow-ups

Participants were contacted via email 1- and 3 months after their treatment sessions to complete the PRPSA and LSAS online.

### Analytic approach

Analyses were performed using R package *brms* 2.9.0^61^ for Bayesian estimation of hierarchical regression models of outcome variables, and *JASP*^62^. We believe that Bayesian estimation is better suited to pilot investigations than typical hypothesis testing, as it produces highly informative parameter estimates for differences between groups/conditions, as well as uncertainty around them, which is not provided by assessments of statistical significance. Uncertainty is in this case expressed as a 95% central posterior density interval (the range between 2.5 and 97.5 percentiles of the posterior). As this was a pilot investigation in which we hoped to indentify a possible effect to focus on in a future randomised controlled trial, formal power analyses were not performed. However, our parameter estimates and model comparison findings suggest that an absence of power does not underpin the null findings: differences of close to zero between conditions were consistently found, and Bayes factors suggested evidence against the inclusion of a propranolol effect. In addition to parameter estimation, we performed cross-validation using the R Package *loo* 2.1.0^40,63^, which can aid in evaluating the predictive value of different experimental variables. Weakly informative default priors in *brm*s were used to analyse questionnaire responses. Physiological analyses did not converge using default priors. We specified slightly more constrained priors, detailed fully in the Supplementary Materials. For analyses of self-report outcome variables, we assessed models, including Session (S1–S2 or S1–3m follow-up), Condition (Placebo vs. Propranolol), Duration (0–9 min) and their interaction. Graphical comparison of change in outcome variables by Session from 0 to 9 min suggested that only a slight linear effect of duration was plausible. Duration was therefore only included as a linear predictor. For physiological outcome measures, Session, Condition, Timepoint (the 3 timepoints noted for cortisol/ambulatory HR collection within each session) and their interaction were included as predictors. Duration was not included, facilitating estimation/interpretation of the three-way categorical interaction. All analyses in *brms* nested repeated measures within participants.

*JASP* was used for analyses of baseline variables, manipulation checks and complimentary computation of Bayes Factors for outcome variables. All *JASP* analyses used default priors outlined by Wagenmakers et al.^64^.

## Supplementary information

Supplementary Regression Tables

## Data Availability

Code for performing the *brms* analyses in *R* can be provided upon request.
