# Supplementary material for: Reconsolidation-based treatment for fear of public speaking: a systematic pilot study using propranolol
Source: Transl Psychiatry. 2020 Jun 4;10:179. doi: 10.1038/s41398-020-0857-z (PMC7272450; doi:10.1038/s41398-020-0857-z)
Supplement: Supplementary file 1 — Supplementary Regression Tables [file 41398_2020_857_MOESM1_ESM.pdf]

Supplementary Materials

Reconsolidation-based treatment for fear of public speaking: A systematic pilot study using propranolol

James W. B. Elsey, Anna I. Filmer, Harriet R. Galvin, Jennifer D. Kurath, Linos Vossoughi,
Linnea S. Thomander, Melissa Zavodnik & Merel Kindt

Medical inclusion/exclusion criteria for propranolol administration

Participants underwent a medical screening to confirm the safe use of propranolol, namely: screening for heart conditions in first degree relatives; a medical history of heart, circulatory, lung, liver, or kidney problems that would contraindicate the use of propranolol; use of contraindicated medications (e.g., use of other medications that affect the heart/blood pressure); heart rate <60, blood pressure <100/60); pregnancy; active asthma. A flow chart of all reasons for exclusion is presented on the following page (Figure S1).

Questionnaire Information

Personal Report of Public Speaking Anxiety (PRPSA: McCroskey, 1970). The PRPSA is a 34-item self-report scale for measuring a respondent's fear of public speaking. Participants respond using a 5-point Likert scale ranging from 1 (strongly disagree) to 5 (strongly agree). Scores can range from 34-170, with higher scores indicating greater PSA. The scale has shown excellent internal consistency (α = .94), with high test-retest reliability (r = .84) making it suitable for repeated measurements (McCroskey, 1970).

Subjective units of distress/discomfort (SUDS, cf. Wolpe & Lazarus, 1966). The SUDS is a brief self-report instrument used to quickly and relatively unobtrusively determine a participant's subjective state of distress. Participants are required to rate their distress from 0 (no distress) to 100 (extreme distress). SUDS scales have shown convergent validity with other measures of distress/anxiety, and also proven sensitive to intervention effects (E. Foa, Riggs, Massie, & Yarczower, 1995; Kim, Bae, & Park, 2008; Soeter & Kindt, 2015; Tanner, 2012).

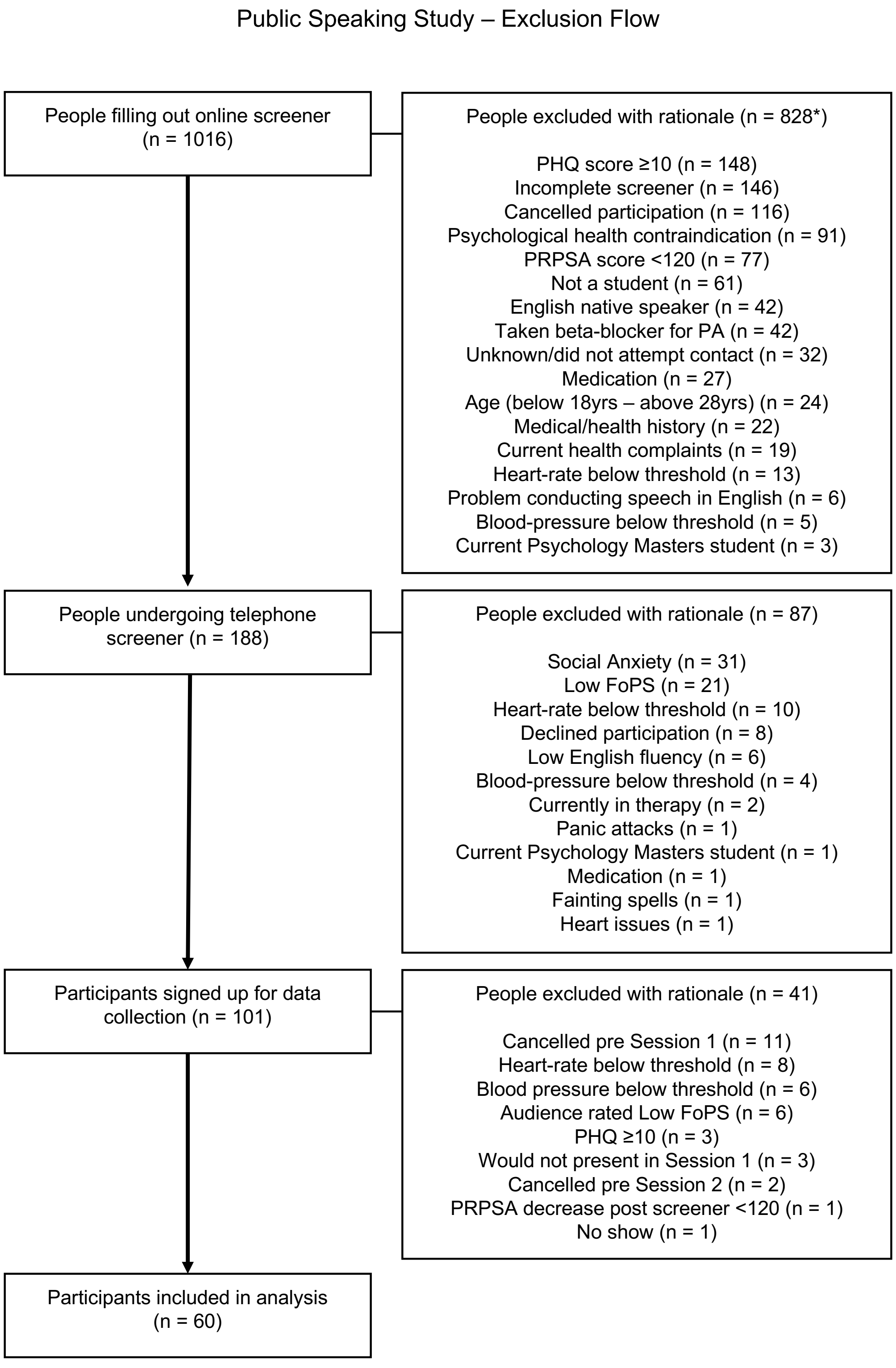

Note. PHQ = Patient Health Questionnaire; PRPSA = Personal Report of Public Speaking Anxiety; FoPS = Fear of public speaking; PA = performance anxiety; * = Reasons contain duplicate for exclusion per candidate

Figure S1. Complete inclusion/exclusion information.

Global Perception of Speech Performance - Self-rating (GPSP: Rapee & Lim, 1992). The GPSP is a brief self-report measure in which respondents assess the general impression they believe they have made upon an audience (e.g., 'Appeared confident'), with items rated from 0 ('Not at all') to 4 ('Very much'). Scores are summed, with higher scores (after reverse scoring items) indicating poorer performance. Internal consistency for the scale is acceptable-to-good (α = .79) (Rapee & Lim, 1992).

Liebowitz Social Anxiety Scale - Self-report (LSAS: Baker, Heinrichs, Kim, & Hofmann, 2002; Heimberg et al., 1999). The LSAS is a self-report scale assessing a respondent's social anxiety. Respondents answer how much fear they would have of (from 0 = "none", to 3 = "severe"), and how often they would avoid (from 0 = "never', to 3 = "usually") 24 social situations. These fear and avoidance subscales have shown good internal consistency (α = .85-.91, Baker et al., 2002; Fresco et al., 2001) and test-retest reliability (r = .79 & .83 respectively, Baker et al., 2002).

Patient Health Questionnaire 9 (PHQ-9: Kroenke, Spitzer, & Williams, 2001). The PHQ-9 is a 9 item self-report scale for assessing depression severity. Participants indicate how much they have experienced 9 depressive symptoms over the past 2 weeks, from 0 ("not at all"), to 3 ("nearly every day"). The scale is deemed a valid measure of depression severity and shows good agreement with diagnoses and other measures of depression (Martin, Rief, Klaiberg, & Braehler, 2006). The official Dutch version of the PHQ-9 was given to Dutch participants.

Anxiety Sensitivity Index (ASI: Reiss, Peterson, Gursky, & McNally, 1986). The ASI is a 16-item self-report scale measuring the degree to which a respondent fears feelings and behaviors associated with anxiety (i.e., anxiety sensitivity). Respondents rate how much they agree with statements reflecting anxiety sensitivity from 0 (very little) to 4 (very much). The robust psychometric properties of the ASI are reported in a review and the scale manual

(Peterson & Plehn, 1999; Peterson & Reiss, 1992). Dutch participants were given a validated Dutch translation of the questionnaire with good internal consistency (α = .83: Vujanovic, Arrindell, Bernstein, Norton, & Zvolensky, 2007).

Rosenberg Self-Esteem Scale (RSES: Rosenberg, 1965). The RSES is a 10 item self-report scale assessing a respondent's sense of self-worth. Respondents indicate the degree to which they agree with statements about satisfaction with their self, from 1 ("strongly disagree") to 4 ("strongly agree"), with higher scores indicating greater self-esteem. The scale has good internal consistency (α = .88, Gray-Little, Williams, & Hancock, 1997) and correlates with related constructs (Schmitt & Allik, 2005). A validated Dutch translation (Franck, De Raedt, Barbez, & Rosseel, 2008) with good internal consistency (α = .86) was used for Dutch participants.

Spielberger State-Trait Anxiety Index (STAI: Spielberger, Gorsuch, & Lusthene, 1970). The STAI is a self-report questionnaire, consisting of two 20-item subscales that measure state (STAI-S) and trait (STAI-T) anxiety. For the STAI-T, respondents answer how frequently they experience anxiety-related phenomena on a scale, from 1 ("almost never") to 4 ("almost always"). For the STAI-S, participants indicate how much they are currently experiencing feelings/thoughts related to anxiety, from 1 ("not at all") to 4 ("very much so"). The subscales have shown good internal consistency (ranging from .83-.92), and the STAI-T shows good test-retest reliability (r = .81) (Foa, Riggs, Dancu, & Rothbaum, 1993). A validated Dutch translation of the STAI was given to Dutch participants (van der Ploeg, 1980).

Saliva analysis procedure

Quantification of salivary analytes was performed by Dresden LabService GmbH. Information on the analysis process was provided by Prof. Dr Clemens Kirschbaum. All samples were kept frozen at -20°c until analysis. Once thawed, salivettes were centrifuged at

3000rpm for 5 minutes, producing a clear, low viscosity supernatant. For cortisol, concentrations were measured using a high sensitivity, commercially available chemiluminescence immunoassay (IBL International, Hamburg, Germany). The intra and interassay coefficients for cortisol were below 7%.

Alpha-amylase concentrations were determined using an enzyme kinetic method. A Genesis RSP8/150 liquid handling system (Tecan, Crailsheim, Germany) was used to process the saliva. This handling system first dilutes (1:625) the saliva with double-distilled water. Twenty microliters of diluted saliva and standard were then transferred to standard transparent 96-well microplates (Roth, Karlsruhe, Germany). "Calibrator f.a.s" solution (Roche Diagnostics, Mannheim, Germany) was used to prepare standard with the following concentrations: 326, 163, 81.5, 40.75, 20.38, 10.19, and 5.01 U/l alpha-amylase. Double distilled ('bidest') water was used as zero standard. A multichannel pipette was then used to pipette 80ml of substrate reagent (alpha-amylase EPS Sys; Roche Diagnostics, Mannheim, Germany) into each well. The microplate with sample and substrate was when incubated in a waterbath for 90 seconds to reach 37°c. Immediately following this, a standard ELISA reader (Anthos Labtech HT2, Anthos, Krefeld, Germany) was used to obtain a first interference measurement, at a 405nm wavelength. A second measurement at 405nm was then taking after incubating the plate in the waterbath at 37°c for another for another 5 minutes. Increases in absorbance were calculated for unknowns and standards. Linear regression calculated for each microplate was used to transform increases of absorbance of diluted samples to alpha-amylase concentrations (Graphpad Prism 4.0c for MacOSX, Graphpad Software, San Diego, CA). Intra and interassay coefficients for amylase were below 9%.

brms physiological prior specification

Analyses of physiological data in *brms* did not converge using default priors. We retained default priors on variance parameters, and specified priors for the Intercept and

predictors that represent a deflection from that intercept. Priors for all such parameters for HR and log-cortisol are presented in Table S1. Log-cortisol values have been multiplied by 10 for ease of specification and reading output.

Table S1. Prior specifications for *brms* physiological analyses

Variable	Parameter	Family	Specification
HR	Intercept	Normal	80, 10
HR	Time = Preparation	Student t	5, 15, 7.5
HR	Time = Speech	Student t	5, 30, 15
HR	Session = S2	Student t	5, 0, 10
HR	Session = S1, Time = Baseline, Placebo	Student t	5, 0, 10
HR	Session = S1, Time = Baseline, Prop	Student t	5, 0, 10
HR	Session = S1, Time = Preparation, Prop	Student t	5, 0, 10
HR	Session = S1, Time = Preparation, Placebo	Student t	5, 0, 10
HR	Session = S1, Time = Speech, Placebo	Student t	5, 0, 10
HR	Session = S1, Time = Speech, Prop	Student t	5, 0, 10
HR	Session = S2, Time = Baseline, Placebo	Student t	5, 0, 10
HR	Session = S2, Time = Baseline, Prop	Student t	5, 0, 10
HR	Session = S2, Time = Preparation, Prop	Student t	5, 0, 10
HR	Session = S2, Time = Preparation, Placebo	Student t	5, 0, 10
HR	Session = S2, Time = Speech, Placebo	Student t	5, 0, 10
HR	Session = S2, Time = Speech, Prop	Student t	5, 0, 10
Cortisol	Intercept	Normal	9, 1.5
Cortisol	Time = Post1	Student t	5, 3, 2
Cortisol	Time = Post2	Student t	5, 3, 2
Cortisol	Session = S2	Student t	5, 0 ,2
Cortisol	Session = S1, Time = Baseline, Placebo	Student t	5, 0 ,2
Cortisol	Session = S1, Time = Baseline, Prop	Student t	5, 0 ,2
Cortisol	Session = S1, Time = Post1, Prop	Student t	5, 0 ,2
Cortisol	Session = S1, Time = Post1, Placebo	Student t	5, 0 ,2
Cortisol	Session = S1, Time = Post2, Placebo	Student t	5, 0 ,2
Cortisol	Session = S1, Time = Post2, Prop	Student t	5, 0 ,2
Cortisol	Session = S2, Time = Baseline, Placebo	Student t	5, 0 ,2
Cortisol	Session = S2, Time = Baseline, Prop	Student t	5, 0 ,2
Cortisol	Session = S2, Time = Post1, Prop	Student t	5, 0 ,2
Cortisol	Session = S2, Time = Post1, Placebo	Student t	5, 0 ,2
Cortisol	Session = S2, Time = Post2, Placebo	Student t	5, 0 ,2
Cortisol	Session = S2, Time = Post2, Prop	Student t	5, 0 ,2

Normal = Mean, SD, Student t = Degrees of freedom, Mean, SD

Note that specified prior families represent distributions of the search space, not for the actual outcome variables. For heart rate, a normal heart rate is between 60-100bpm (https://www.heart.org/en/health-topics/high-blood-pressure/the-facts-about-high-blood-pressure/all-about-heart-rate-pulse#.Wg1mcBO0OCU). We set the Intercept to 80 with an SD of 10, as values very close to 60 are also unlikely given that this is set as a lower bound for study inclusion. All other priors use a *t* distribution with 5 degrees of freedom. The *t* distribution is similar to the normal, only having larger tails (coming closer to the normal distribution as degrees of freedom increase). Use of this distribution means that we can set reasonable bounds on the probable parameter values, but allow the search space to consider large values should they arise in the data. In reviewing 10 years of research on the TSST, Kudielka, Hellhammer, & Kirschbaum (2007) indicate that HR can be expected to increase from between 15-25bpm. Given that we are investigating anxious individuals and taking the first minute of their speech where HR can be expected to be higher, we chose a slightly higher value of 30 with standard deviations and degrees of freedom that allow much lower or higher values to be considered. We select half this value for the preparation period. For all interaction terms, the mean is set to 0, with degrees of freedom and standard deviations enabling potentially sizable interactions to be accepted, while considering a null effect as most plausible.

For cortisol, we used approximate starting values for cortisol levels in previous studies (e.g., Kirschbaum, Pirke, & Hellhammer, 1993), which seem to be between 6 and 10. Log 8 is 0.9, so we take this as a starting point for the intercept. Kudielka et al. (2007) suggest that typical responses in women (which predominate in our sample) are for cortisol increases of 50-150%. Doubling 8 gives 16, and log 16 is 1.2, giving a difference on the log scale of 0.3, which we thus take as the likely deviation around which to search for values in the post-stress period. Again we give sufficient degrees of freedom and standard deviations

such that considerably higher or lower values will be considered. As with HR, we set interactions to a mean of 0, with degrees of freedom and standard deviations allowing sizable positive or negative effects, with the null being given the most initial plausibility.

Manipulation check

Table S2. Means for manipulation check

Variable	Time	Condition	Mean (SD)	n
HR	Start	Placebo	70.89 (6.71)	20
		Propranolol	71.11 (10.75)	40
	End	Placebo	62.67 (7.22)	20
		Propranolol	55.27 (8.22)	40
$BP_{Systolic}$	Start	Placebo	117.1 (6.67)	20
		Propranolol	117.9 (10.15)	40
	End	Placebo	111.9 (6.70)	20
		Propranolol	108.1 (9.33)	40
$BP_{Diastolic}$	Start	Placebo	72.58 (13.67)	20
		Propranolol	73.43 (8.16)	40
	End	Placebo	71.15 (6.93)	20
		Propranolol	67.18 (9.83)	40
Amylase	Start	Placebo	1.83 (0.35)	20
		Propranolol	1.87 (0.41)	37*
	End	Placebo	1.71 (0.35)	20
		Propranolol	1.44 (0.32)	37*
STAI-S	Start	Placebo	40.25 (9.68)	20
		Propranolol	43.55 (8.61)	40
	End	Placebo	30.95 (7.72)	20
		Propranolol	31.65 (6.02)	40

* saliva/amylase could not be assayed from 3 samples

Table S2. $BF_{Inclusion}$ of Session (S), Condition (C), Duration (D), and their interaction (*).

		$BF_{Inclusion}$					
		PRPSA	$LSAS_{Fear}$	$LSAS_{Avoid}$	Distress (Ant)	Distress (Max)	GPSP
RM ANOVA	S	7.08e +8	7424.54	26.1	1099.51	2.64e +8	2.54e +9
	C	0.29	0.45	0.47	0.39	0.70	0.35
	S*C	0.11	0.11	0.28	0.35	0.50	0.33
Regession on change scores	D	0.22	0.20	0.24	0.20	2.35	0.61
	C	0.19	0.22	0.31	0.18	0.53	0.32
	C*D	0.19	0.24	0.34	0.19	0.60	0.35

RM ANOVA = Bayesian repeated measures ANOVA; $BF_{Inclusion}$ = Bayes factor for inclusion of respective model component, Ant = Anticitpatory distress

Ordered probit models for questionnaire responses

Recent discussions argue that most questionnaires, being aggregations of ordinal Likert items, are not optimally analysed using metric models (1, 2). We thus additionally analysed GPSP and PRPSA responses using an ordered probit model in *brms*, to supplement the standard analyses. These models additionally tested the inclusion of a varying slope for the effect of Session across participants.

Results of cross-validation analyses for these probit models are presented in Figure S2. Corroborating the key take-aways from the more typical regression analyses, ordered probit models for GPSP and PRPSA items similarly indicate that adding Session improves predictions, with further predictors yielding negligible gains. Including a varying impact of Session across participants further improves model predictions, again with no evidence favoring additional predictors.

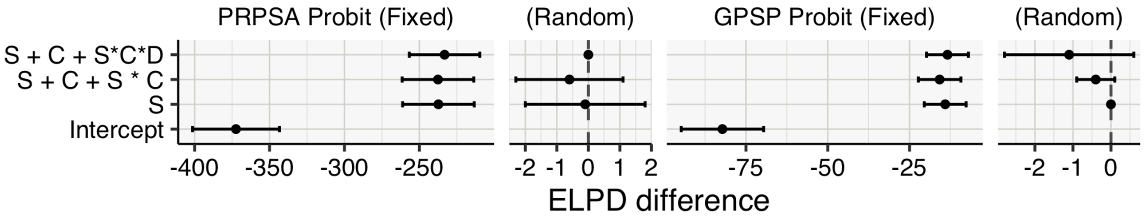

Figure S2. Model performance in leave-one-out cross validation for each primary and secondary outcome variable, indicating improvement of model performance with the inclusion of Session and no benefit of additional predictors. ELPD = expected log pointwise predictive density vs. best model. (Fixed) = probit model with fixed effects of session; (Random) = probit model with random/varying effects of session. S = Session, C = Condition, D = Duration, * = interaction between predictors.

Posterior summaries of regression models from brms

The large number of tables here takes considerable space. It is available as a data supplement (a separate excel document) and will further be uploaded to the Open Science Framework where it can be downloaded.

Fitted means for Physiological Analyses

Analyses of physiological outcome variables indicated that timepoint was the only meaningful predictor for HR, and no predictors were sufficiently informative be included in a model for log-cortisol responses. We nevertheless report in Table S4 the fitted means (estimates using the regression equation and posterior parameter estimates) in each condition at each time point, for HR and cortisol, for the Session*Condition*Timepoint model.

Table S4. Fitted means for physiological outcomes

Variable	Condition	Session	Timepoint	Mean [95% PDI]
HR	Placebo	S1	Baseline	71.23 [65.27-77.20]
HR	Propranolol	S1	Baseline	71.33 [66.93-75.70]
HR	Placebo	S1	Preparation	89.76 [83.89-95.80]
HR	Propranolol	S1	Preparation	93.53 [89.10-97.83]
HR	Placebo	S1	Speech	108.40 [102.15-114.66]
HR	Propranolol	S1	Speech	116.45 [111.92-120.85]
HR	Placebo	S2	Baseline	72.13 [66.23-78.22]
HR	Propranolol	S2	Baseline	74.76 [70.35-79.18]
HR	Placebo	S2	Preparation	89.93 [83.98-95.90]
HR	Propranolol	S2	Preparation	94.90 [90.44-99.31]
HR	Placebo	S2	Speech	110.92 [104.87-117.05]
HR	Propranolol	S2	Speech	117.90 [113.45-122.39]
Cortisol	Placebo	S1	Baseline	0.731 [0.619-0.846]
Cortisol	Propranolol	S1	Baseline	0.758 [0.674-0.841]
Cortisol	Placebo	S1	Post 1	0.748 [0.634-0.864]
Cortisol	Propranolol	S1	Post 1	0.789 [0.707-0.871]
Cortisol	Placebo	S1	Post 2	0.810 [0.695-0.924]
Cortisol	Propranolol	S1	Post 2	0.818 [0.733-0.900]
Cortisol	Placebo	S2	Baseline	0.725 [0.611-0.839]
Cortisol	Propranolol	S2	Baseline	0.776 [0.691-0.859]
Cortisol	Placebo	S2	Post 1	0.746 [0.631-0.861]
Cortisol	Propranolol	S2	Post 1	0.883 [0.801-0.967]
Cortisol	Placebo	S2	Post 2	0.718 [0.604-0.831]
Cortisol	Propranolol	S2	Post 2	0.906 [0.822-0.989]

Supplementary References

1. Liddell TM, Kruschke JK. Analyzing ordinal data with metric models: What could possibly go wrong?. Journal of Experimental Social Psychology. 2018 Nov 1;79:328-48.

2. Bürkner PC, Vuorre M. Ordinal regression models in psychology: a tutorial. Advances in Methods and Practices in Psychological Science. 2019 Mar;2(1):77-101.
